# Assessment of Primary Health Care Nurses’ Knowledge of Forensic Nursing Competencies

**DOI:** 10.1590/0034-7167-2025-0319

**Published:** 2026-03-30

**Authors:** Jhuliano Silva Ramos de Souza, Zélia Marilda Rodrigues Resck, Rogério Silva Lima, Fábio de Souza Terra, Thiago Augusto Soares Monteiro da Silva, Taciana Silveira Passos, Sueli de Carvalho Vilela

**Affiliations:** I Universidade Federal de Alfenas. Alfenas, Minas Gerais, Brazil; II Universidade de Vassouras. Vassouras, Rio de Janeiro, Brazil; III Universidade de Brasília. Brasília, Distrito Federal, Brazil

**Keywords:** Forensic Nursing, Primary Health Care, Education, Nursing, Professional Competence, Knowledge., Enfermería Forense, Atención Primaria de Salud, Educación en Enfermería, Competencia Profesional, Conocimiento.

## Abstract

**Objectives:**

to analyze the knowledge of Primary Health Care nurses regarding Forensic Nursing competencies before and after an educational intervention.

**Methods:**

a quasi-experimental, before-and-after study was conducted with 12 professionals from the southern region of Minas Gerais between March and August 2023. The intervention was developed using the Problematization Methodology, and knowledge was assessed using an instrument developed by the researchers. The analysis was performed using descriptive statistics and Fisher’s exact test.

**Results:**

an increase in knowledge was observed in categories related to violence, crime, legislation, ethics, mental disorders, and disaster response. The training improved identification and intervention in various forms of violence, although gaps remained in topics such as forensic patient care and evidence preservation.

**Conclusions:**

continuing education is necessary to enhance knowledge in critical areas of Forensic Nursing practice.

## RESUMEN

**Objetivos:**

analizar el conocimiento de los enfermeros de Atención Primaria de Salud sobre competencias en Enfermería Forense, antes y después de una intervención educativa.

**Métodos:**

estudio cuasi experimental del tipo antes y después, realizado con 12 profesionales de la región sur de Minas Gerais entre marzo y agosto de 2023. La intervención se llevó a cabo mediante la Metodología de la Problematización y un instrumento creado por los investigadores para evaluar los saberes. El análisis se realizó mediante estadística descriptiva y prueba exacta de Fisher.

**Resultados:**

se observó un aumento del conocimiento en las siguientes categorías: violencia, delincuencia, legislación, ética, trastornos mentales y respuesta ante desastres. La capacitación mejoró la identificación y la intervención en diversas formas de violencia, aunque existían lagunas en temas como la asistencia a pacientes forenses y la preservación de pruebas.

**Conclusiones:**

la formación continua es necesaria para mejorar los conocimientos en áreas críticas de la práctica de la Enfermería Forense.

## INTRODUCTION

Forensic Nursing, recognized as a specialty in Brazil since 2011^(1)^ and regulated by the Federal Council of Nursing^(2)^, integrates nursing care and forensic practices in the care of victims, perpetrators, and their families in contexts of violence and crime^(3)^. The Competency Matrix highlights eight main areas, such as sexual violence, the prison system, psychiatry, evidence collection, postmortem, forensic and consulting, mass disasters, and abuse throughout the life cycle^(2)^.

The specialty encompasses 29 general competencies distributed across six areas, such as assessment and intervention in violence and trauma, acting as a forensic expert, applying the Nursing Process to the prison population, and disaster response and humanitarian missions, with a focus on interdisciplinary collaboration. Furthermore, it includes 37 specific competencies, such as health risk assessment for victims of sexual violence, assistance in the criminal justice and psychiatric systems, report preparation, preservation of remains, photographic documentation, and post-mortem care in disasters^(2)^.

These professional competencies are based on ethical and legal aspects and on assistance to people in situations of violence^(4,5)^. Violence is a serious public health problem and can manifest itself in various forms, including physical, psychological and/or emotional, domestic, and sexual^(6)^. It is essential that healthcare professionals be prepared to identify and report cases through the Notifiable Injuries Information System (NIIS)^(7)^.

Nursing in Primary Health Care (PHC) is vital for recognizing signs and symptoms of violence, but many professionals lack the training and skills to manage these situations^(8)^. The lack of mandatory reporting and the need for training to serve these individuals are critical issues that demand educational strategies and effective public policies^(9)^. Educational interventions, especially in professional and academic settings, are essential to prepare future healthcare professionals to deal with violence^(10,11)^. Training should include specific content on violence, enabling nurses to recognize and intervene in different cases of violence and other forms of aggression in the forensic setting, as effective strategies for combating and preventing this global phenomenon^(12)^.

The scarcity of studies on forensic nursing in Brazilian educational institutions highlights the need to incorporate these topics into academic training to promote more effective evidence-based clinical practice^(13,14)^. The work of forensic nurses is essential for identifying and intervening in cases of violence. Assessing these professionals’ knowledge of their professional competencies is essential to improving the care provided, in addition to contributing significantly to other areas, such as justice, education, and public safety^(15)^.

### Significance of the study

This study is an excerpt from the doctoral thesis of one of the authors, titled Forensic nursing competencies: assessment of nurses’ knowledge^(16)^.

## OBJECTIVES

To analyze the knowledge of Primary Health Care Nurses regarding Forensic Nursing competencies before and after an educational intervention.

## METHOD

### Ethical aspects

The study was approved by the university’s Research Ethics Committee. Participants were invited to participate via email and, if they agreed, were automatically redirected to the electronic Informed Consent Form (ICF), in accordance with Resolution No. 466/12.

### Study design, period and setting

This intervention study presents characteristics of a quasi-experimental research, using preand post-tests without a control group. It was conducted with PHC nurses from the southern region of Minas Gerais. Data collection took place between March and August 2023.

### Population or sample; inclusion and exclusion criteria

The total number of participants working in PHC was 153 professionals, 125 of whom were affiliated with the Family Health Strategy (FHS) and 28 with Basic Health Units (BHU). Ninety-six professionals were recruited for the study, of whom 42 participated in the pre-test and 12 completed all stages of the research (pre-test, intervention, and post-test), comprising the final study sample. All participants had at least one year of experience, were working during the research period, and were available to participate in all three study phases. Exclusion criteria included those on vacation and/or on medical leave.

### Study protocol

The study was conducted in three stages: pre-test, intervention, and post-test. The first stage assessed the profile and prior knowledge using the “Forensic Nursing Knowledge Assessment Questionnaire in PHC”, developed and validated by the authors^(17)^. It is self-administered, Likert-type, with five answer options. The questionnaire was administered via Google Forms before and after the intervention.

The second stage involved the educational intervention, which consisted of a theoretical-practical extension course based on the Competency Matrix^(3)^. It was structured into eight modules: I: Contextualization of the specialty; II: Main areas of practice in Brazil; III: Typologies of violence and their characteristics; IV: Forensic Nursing Process; V: Role of Nursing in Forensic Evidence; VI: Forensic Aspects in Psychiatric Situations; VII: Abuse, Trauma, and Other Forms of Violence; and VIII: Forensic Nursing and Civil and Criminal Rights. The course was taught simultaneously by the three researchers. The professionals received the basic content delivered digitally. The course load consisted of 60 hours, 40 of which were synchronous and 20 asynchronous. The Problematization Methodology with the Maguerez Arc was used as a pedagogical structure^(18)^. It was taught online, via Google Meet, synchronously, with 10 weekly meetings of four hours each, on Tuesdays. Asynchronous activities were carried out on Wednesdays using tools such as Google Forms and Moodle. Furthermore, participants received a handout with theoretical content and case studies. Finally, in the third session, after the intervention, participants were reassessed using the aforementioned questionnaire^(17)^.

### Analysis of results and statistics

For analysis and organization, we used the R software, version 4.2.3. Participants’ sociodemographic and employment characteristics were characterized through descriptive and inferential analyses, both in the pre-test and post-test. Knowledge of area competencies was assessed using descriptive statistics and Fisher’s exact test, with a 5% significance level for preand post-extension course comparisons.

## RESULTS

The analysis of sociodemographic data revealed a predominance of females in both stages (90.48% in the pre-test and 75.00% in the post-test), with a higher concentration (57.14% in the pre-test and 50.00% in the post-test) aged between 30 and 40 years. A change was observed in the color/race variable, with a brown majority in the pre-test (69.05%) and white in the post-test (75.00%). Regarding marital status, married/partnered individuals were the majority in the pre-test (57.14%), while single individuals predominated in the post-test (50.00%). There was an increase in education: 19.05% had completed higher education in the pre-test, against 100% in the post-test, with 83.33% pursuing postgraduate studies. Family income remained stable between 4 and 6 minimum wages (52.38% in the pre-test and 50.00% in the post-test). Catholicism prevailed in both phases (76.19% in the pre-test and 83.33% in the post-test).

Regarding the work profile, the distribution by municipality varied, with Alfenas and Muzambinho cities maintaining participation in both phases. Professional experience between 5 and 10 years increased from 14.29% to 50%, indicating a greater participation of professionals with established experience. The workload was predominantly 8 hours per day (97.62% in the pre-test and 100% in the post-test), and the majority did not have another job. Knowledge assessments between the preand post-test revealed significant variations in competencies related to Forensic Nursing, as detailed below.

### A) Physical, Psychological and/or Emotional, Sexual and Domestic/Family Violence

The educational intervention demonstrated an impact on knowledge regarding the different types of violence - physical, psychological/emotional, sexual, and domestic/family. Regarding physical violence, an association was observed in identification (p<0.0216), assessment, and intervention (p<0.0063), with an increase of up to 80.00% in knowledge after the course. Regarding psychological/emotional violence, there were advances in identification (p<0.0074), with 75.00% assertiveness, and in intervention (p<0.0127), with 90.00% of professionals reporting greater confidence, although 16.00% still presented uncertainty in the assessment. Sexual violence also showed an association, with increased knowledge for identification (p<0.0012), assessment (p<0.0359), and intervention (p<0.0095), with approximately 90.00% of professionals feeling more prepared after the training. Similarly, data on domestic/family violence indicated an association in identification (p<0.0024), assessment, and intervention (p<0.0127), with 90.00% of participants expressing greater confidence in acting. Overall, the results confirm that the training significantly strengthened professionals’ skills, especially in the areas of identification and intervention, highlighting the importance of educational strategies to improve performance in the face of different forms of violence.

### B) Forensic Nursing-Related Crime

Regarding the identification of situations associated with crime, the results showed an association in the identification of perpetrators of violence (p<0.0007), individuals with violent behavior (p<0.0060), attempted or completed illegal abortions (p<0.0009), and victims of violence within the prison system (p<0.0043). Most professionals (50.00%) reported increased confidence and preparedness after the intervention, demonstrating that the course expanded their perception and ability to recognize these critical situations.

Regarding the evaluation of forensic contexts, before the training was offered, professionals reported having no knowledge on the topic. After the intervention, there was an improvement in knowledge (40.00%) regarding the performance of forensic examinations at the Forensic Medical Institute (IML), with an association in the post-test (p<0.0006). Despite the progress, the evaluation indicated a need for deeper exploration of some content, especially those involving greater technical and practical complexity.

Furthermore, the training was effective in expanding knowledge of procedures for various areas (40.00%). An association was observed in variables related to forensic examination at the IML (p<0.0031) and in the collection (p<0.0047), storage (p<0.0051), and disposal of biological material (p<0.0043). The majority (50.00%) demonstrated greater mastery of the correct storage of these materials, indicating a positive impact, although the data also indicate that practices with greater operational complexity still require reinforcement.

The management of trace evidence related to the cause of death (50.00%) also showed progress, with associations in the stages of collection (p<0.0033), collection (p<0.0007), and documentation of evidence (p<0.0019). The results suggest that the modules on this topic were effective; However, given the practical nature of the skills, exclusively remote learning may have limited some participants’ self-confidence, thus requiring more interactive teaching strategies.

Other areas of intervention that demonstrated knowledge growth included: managing perpetrators in the prison system (p<0.0014), working with people in custody (p<0.0028), referrals to specific agencies (p<0.0112), and filing police reports (p<0.0185). Although the gains were evident (60.00%), it is recommended that content on legal procedures and institutional workflows be reinforced to ensure greater professional mastery.

Finally, the training demonstrated results in the preparation of expert reports (p<0.0003) and reporting cases to NIIS (p<0.0127), the latter being the area that showed the least progress (75.00%). This suggests that, despite the impact of the intervention, content related to epidemiological surveillance and official case registration requires more practical approaches and examples applied to the reality of PHC.

### C) Brazilian Legislation Regarding Forensic Nursing

The results indicated an association with increased knowledge of specific social protection legislation (66.00%). There was an improvement in understanding of the Maria da Penha Law (p<0.0109), the Feminicide Law (p<0.0059), the Menino Bernardo Law (p<0.0001), the Child and Adolescent Statute (ECA) (p<0.0088), and the Next Minute Law (p<0.0009). These data indicate that professionals expanded their ability to recognize the legal provisions that support the identification and referral of cases of violence within PHC.

There was also an association in knowledge of other legal regulations that impact practical work (58.00%), such as the Joanna Maranhão Law (p<0.0001), which addresses child and adolescent victims of violence. Compulsory reporting of violence against women (VAW) also showed an improvement in understanding (p<0.0037), reinforcing the importance of professionals as agents of surveillance and protection.

Additionally, 66.00% of participants demonstrated increased knowledge of the Elderly Statute (p<0.0205), essential legislation for defending the rights of a growing and often vulnerable population, as well as the role of nurses as judicial experts, with association (p<0.0001), highlighting the impact of the course on expanding understanding of the specialty’s specific legal responsibilities.

### D) Ethics and Bioethics in Forensic Nursing

Regarding the identification and evaluation of ethical behaviors such as recklessness, incompetence, negligence, and mistreatment, the course promoted advances mainly in the identification of recklessness (p<0.0328) and its evaluation (p<0.0037), indicating greater clarity among participants regarding the limits of safe action. However, although responses improved in questions related to the identification of incompetence (75.00%), negligence, and mistreatment, there was an association suggesting that the content may have generated reflection, but not enough to significantly change the level of knowledge on these topics in the group evaluated. On the other hand, in the evaluation of these same behaviors (65.00%), associations were found in incompetence (p<0.0072), negligence (p<0.0078), and mistreatment (p<0.0078), demonstrating a greater understanding of the consequences of these technical and behavioral failures in professional practice. Regarding legal and ethical foundations, participants showed improvement (66.00%) in their knowledge of the forensic medical process (p<0.0043), the professional code of ethics (p<0.0183), professional secrecy (p<0.0308), and general concepts of ethics and bioethics (p<0.0205). The association between these variables demonstrates that the course was effective in promoting a more solid understanding of the principles that govern nursing conduct, especially in the management of sensitive and legally relevant situations.

### E) Mental and Behavioral Disorders Related to Forensic Nursing

The training provided advances in knowledge (75.00%) regarding mental health, violent behavior, suicide attempts, and psychoactive substance use-topics often undervalued in daily clinical practice. There was a correlation between assessment (p<0.0154) and intervention (p<0.0068) in cases of violence directed at oneself or others.

Regarding suicide, improvements were identified in all stages analyzed (75.00%), with associations in identification (p<0.0400), assessment (p<0.0246), and intervention (p<0.0068). Regarding the use and abuse of alcohol and other drugs, participants showed improvements in assessment (p<0.0328) and intervention (p<0.0068), demonstrating greater confidence in dealing with these cases after the course. For individuals with violent behavior, agreement in identification increased from 50% to 83.33%, and in intervention, from 41.67% to 58.33%, both with a correlation. A similar situation was observed for perpetrators with mental disorders, whose agreement rates increased from 33.33% to 75% in identification and to 58.34% in intervention, with p-values also significant (p<0.05).

In the care provided to individuals with mental disorders under court order, a decrease in neutral responses and an increase in agreement were notable, demonstrating greater clarity on the part of professionals, although the improvement was more modest. In cases of compulsory hospitalization, progress was observed in identification (from 41.67% to 58.33%) and intervention (from 33.33% to 41.67%), with associations that reinforce the effectiveness of the training (p<0.05).

### F) Mass Disasters, Catastrophes and Humanitarian Missions

The training also covered urgent and emergency situations involving automobile accidents, drownings, mass disasters, gunshot wounds, and asphyxiation. A statistically significant association (p<0.05) was observed in the stages of identification, assessment, and understanding of forensic concepts, indicating greater confidence and preparedness of professionals to work in these contexts (80.00%). Regarding referrals to services such as the Mobile Emergency Care Service, Fire Department, and Emergency Room, approximately 90.00% of nurses already demonstrated prior mastery, a trend maintained in the post-test. Despite the slight variation, the data (p<0.0431) suggest that the course served as an important reinforcement, consolidating already established practices and strengthening the performance of PHC nursing in emergency scenarios with forensic implications.

### G) Interpersonal Relationship with Victims and Perpetrators

The training covered topics related to humanized care, emotional support, and Unconditional Positive Regard (UCR), especially in critical contexts such as death, disasters, and suffering. Family support regarding the cause of death (p<0.0370) and a non-judgmental stance showed little variation between tests, indicating consolidated prior knowledge (80.00%). On the other hand, a significant improvement was observed in emotional support for disaster victims (p<0.0067), demonstrating that the course filled initial gaps. There was also progress in support for perpetrators (p<0.05), although this area still requires further development due to the complexity and stigma involved. Regarding the UCR, the results demonstrated progress (p<0.0059), reinforcing that the training contributed (66.00%) to increasing ethical awareness and professional preparation, even though the practice still requires institutional support and ongoing training to be fully implemented.

## DISCUSSION

The findings demonstrate the impact of the intervention, addressing types of violence, crime, forensic aspects, legislation, mental and behavioral disorders, mass disasters, humanitarian missions, and interpersonal relationships. Research conducted in Brazil^(19,20)^ and Portugal^(21)^ highlights the importance of training programs that address forensic practice in its multiple dimensions, contributing to a more solid professional education. This translates into more effective interventions when serving populations affected by violence. Expanding knowledge about competencies is essential for these professionals to act more effectively in critical situations^(22)^. This initiative is aligned with the guidelines of the World Health Organization (WHO)^(6)^, which recognizes violence as a serious public health problem affecting children, women, and the elderly.

The sociodemographic and occupational profile reveals that nursing in Brazil is predominantly female and includes a significant proportion of Black and brown professionals, in addition to being marked by married professionals, with Catholicism as the predominant religion^(23,24)^. A study conducted in the Federal District indicates that most of these professionals have worked in PHC for over 12 years^(23)^. This sociodemographic configuration may impact the assimilation and application of acquired knowledge, as it indicates that women in this age group are more sensitive to issues related to violence^(24)^.

The training demonstrated a positive impact, reflected in increased knowledge about intervention in cases of violence. These results are consistent with a study conducted in Iran^(25)^, which highlighted the effect of educational programs on professionals’ ability to develop appropriate strategies to improve knowledge and clinical decision-making when dealing with forensic patients. The intervention addressed different types of violence, favoring more assertive action in both identifying and responding to the situations presented. This approach also aligns with the findings of a study conducted in India^(26)^, which emphasizes the importance of training professionals to work and improve forensic care.

On the other hand, a study revealed challenges faced by professionals in assisting women experiencing violence. The main problems identified were a lack of training, which results in service failures and inadequate care, in addition to underreporting of cases^(27)^. The predominant approach observed was skilled listening, considered an essential skill in caring for victims of violence^(27)^.

The literature on interventions in cases of physical violence indicates that women are the main victims^(28)^. Professionals are underprepared, not only facing challenges in treating physical injuries but also lacking a broader approach that considers the psychological aspects of violence. Interventions should include welcoming, identifying signs and symptoms, and referring to support networks that integrate interdisciplinary teams. To improve this situation, it is essential to invest in continuing education in services and include courses that address this topic in teaching curricula^(28,29)^. Regarding the assessment and intervention of psychological and/or emotional violence, a study conducted in South Africa^(30)^ and another in Australia^(31)^ reveal that nurses working in mental health and forensic psychiatry services face different forms of violence, including violence that generates physical and psychological trauma. The aforementioned studies highlight that this exposure has a negative psychological effect on professionals, affecting their mental health. To cope with these experiences, professionals use different strategies, and the study suggests that offering psychological support and specific training can help them better manage the impact of the violence they face in the professional environment.

Regarding intervention in sexual violence, the data from this study showed an impact with participants reporting agreement regarding the intervention after the course. This result is relevant, since sexual violence represents one of the greatest challenges for professionals, due to its complexity and ethical, legal, and clinical implications^(32)^. The research also highlights the weaknesses in professional training, evidenced by the insufficiency of current training measures, which often leaves professionals feeling unprepared to deal with cases of sexual crimes^(33)^.

In the context of intervention and assessment in cases of domestic and/or family violence, the course promoted advances, evidenced by increased knowledge about appropriate interventions. A study on forensic work with elderly people in situations of violence^(34)^ emphasizes that generalist nurses often apply specialty strategies, even without formally recognizing this knowledge. However, barriers exist that hinder this care, with the lack of adequate training being one of the main difficulties^(34)^.

In addition to strengthening knowledge about crime and forensic practices, the intervention contributed to the understanding of topics such as illegal abortion, assistance to the perpetrator, and forensic procedures. Despite advances in the collection and storage of biological material, no association was observed, indicating the need for more in-depth and targeted training on this topic. When well-received by PHC professionals, the intervention favors early identification and appropriate referral of cases, both within and outside the network, increasing the visibility and effectiveness of violence care services. However, its implementation still faces challenges, related to the organizational structure of municipal services and the prevailing care culture, which can limit the full incorporation of forensic practices into PHC routines^(35)^.

According to the New Zealand study^(36)^, one of the greatest challenges faced is the practical application of forensic techniques, which may explain the results obtained. Furthermore, the study revealed limited knowledge about forensic patient care, evidence preservation and collection, legislation, and/or legal processes. The suggestions indicate that forensic education is necessary and desired among professionals within the clinical environment. A study developed in Brazil^(19)^ highlights the need for knowledge, training, and practical participation of professionals qualified to provide care and collect evidence for legal purposes in the care of patients who are victims of violence.

The impact of training on legislative knowledge in the forensic field, including laws such as the Maria da Penha and the Feminicide Act. Brazilian legislation^(37)^ on the protection of women has gained prominence in discussions on this topic. There is a need to include, from continuing education and team training, knowledge of the standards that guarantee protection for women in situations of violence. This understanding must be broadly assimilated by professionals, enabling the development of effective prevention and protection actions. A lack of this knowledge can limit the appropriate performance of professionals facing this reality^(38)^.

Training has also proven effective in improving knowledge of ethics and bioethics and the association between these variables. This area is frequently cited as a challenge in forensic practice, as it involves sensitive aspects such as preserving confidentiality and the ethical conduct of criminal cases. A study conducted in India^(26)^ highlights that the role of nursing goes beyond technical-scientific competencies. When collecting evidence, forensic nurses must act without prejudice, with caution, and with attention to preventing and reducing violence. Furthermore, it is essential to develop ethical, legal, and cultural skills, ensuring that no harm is caused to the person in a criminal situation.

Although the training increased knowledge about identifying and intervening in cases of mental and behavioral disorders, the results were significant. This finding may be related to the complexity of cases involving mental health and forensic care, as highlighted in a study conducted in Japan^(39)^, in which researchers observed that in many cases, forensic psychiatric nurses face difficulties managing these cases without continuous support, lack of specialized training, and neglected care due to the lack of interventions that could change this reality.

The results related to training for mass disasters and humanitarian missions indicate an increase in knowledge and engagement. This result is consistent with studies that point to the need for greater emphasis on training in disasters and emergencies, areas in which nurses often lack specific training^(20)^. Preparedness for extreme situations is crucial, as also highlighted in the literature on disaster response^(19)^. The analysis of interpersonal relationships with victims and perpetrators highlighted the positive pre-existing knowledge among nurses, with an increase in knowledge about emotional support after training. Although the results were significant, this aspect reinforces the importance of training focused on communication and empathy, fundamental areas for forensic nursing practice in the humanitarian context^(40)^.

Furthermore, nurses, as the largest group of caregivers and in constant contact with people in situations of violence, play a crucial role in defending these individuals, especially in preserving evidence. Therefore, it is essential to invest in the training of these professionals for more comprehensive and effective action, contributing to optimal care for this population^(41)^.

Therefore, violence is a persistent problem in contemporary society, and forensic nurses play a crucial role in addressing it, as they possess specialized knowledge to work in criminal cases. Forensic practice encompasses not only the analysis of victims and perpetrators, but also assistance to survivors and family members, highlighting its importance in a comprehensive response to this phenomenon^(42)^.

### Study limitations

Among the study’s limitations is the small sample size, which limits the generalizability of the results. Furthermore, PHC professionals had difficulty understanding some topics, especially forensic science, legislation, and mass disasters. Despite the online course offering weekly meetings, the topic may have been perceived as distant from daily PHC practice. Although the course covers basic concepts, significant gaps remain in areas such as care for perpetrators and legal action. These findings reinforce the need for continuous and structured forensic content in professional training to enhance their ethical, technical, and legal response capabilities in more complex situations.

### Contributions to the field of Nursing, Health or Public Policy

Forensic nursing competencies contribute significantly to combating violence by empowering primary care professionals to identify signs of abuse, preserve evidence, and act ethically and legally. This practice strengthens the integrated response to violence in health services, improves victim care, and supports more effective and humane public policies, promoting positive impacts on community health and social protection.

## CONCLUSIONS

The analysis of PHC nurses’ knowledge of forensic nursing, conducted in three stages (pre-test, intervention, and post-test), demonstrated significant progress after the training. There was an improvement in their skills in identifying, assessing, and intervening in various forms of violence, as well as a greater understanding of criminality, ethics, bioethics, and the legal aspects of forensic practice. Participants also demonstrated greater preparedness to work in complex contexts such as disasters, mental disorders, and emergency situations with legal implications. However, gaps remain in topics such as forensic patient care, evidence management, and technical-legal knowledge. These findings highlight the need for ongoing educational programs focused on the forensic training of PHC professionals, aligned with the current requirements of ethical and legal practice in primary care.

## Data Availability

Not applicable.
