# Supplementary material for: Assessment of Primary Health Care Nurses’ Knowledge of Forensic Nursing Competencies
Source: Rev Bras Enferm. 2026 Mar 30;79:e20250319. doi: 10.1590/0034-7167-2025-0319 (PMC13035285; doi:10.1590/0034-7167-2025-0319)
Supplement: Translation

# Translation: Avaliação do Conhecimento dos Enfermeiros da Atenção Primária à Saúde sobre Competências em Enfermagem Forense

**DOI:** 10.1590/0034-7167-2025-0319pt

**Authors:** Jhuliano Silva Ramos de Souza, Zélia Marilda Rodrigues Resck, Rogério Silva Lima, Fábio de Souza Terra, Thiago Augusto Soares Monteiro da Silva, Taciana Silveira Passos, Sueli de Carvalho Vilela

**Affiliations:** I Universidade Federal de Alfenas. Alfenas, Minas Gerais, Brasil; II Universidade de Vassouras. Vassouras, Rio de Janeiro, Brasil; III Universidade de Brasília. Brasília, Distrito Federal, Brasil

**Keywords:** Enfermagem Forense, Atenção Primária à, Saúde, Educação em Enfermagem, Competência Profissional, Conhecimento.

## Abstract

**Objetivos:**

analisar o conhecimento dos enfermeiros da Atenção Primária à Saúde sobre competências em Enfermagem Forense antes e após uma intervenção educativa.

**Métodos:**

estudo quase experimental do tipo antes e depois, realizado com 12 profissionais da região do Sul de Minas Gerais entre março e agosto de 2023. A intervenção foi desenvolvida pela Metodologia da Problematização, e o conhecimento foi avaliado através de um instrumento elaborado pelos pesquisadores. A análise foi realizada por meio de estatística descritiva e o teste exato de Fisher.

**Resultados:**

observou-se um aumento do conhecimento nas categorias relacionadas à violência, criminalidade, legislação, ética, transtornos mentais e resposta a desastres. A capacitação melhorou a identificação e intervenção em diversas formas de violência, embora ainda tenha havido lacunas em tópicos como assistência a pacientes forenses e preservação de evidências.

**Conclusões:**

a formação continuada é necessária para aprimorar o conhecimento em áreas críticas da prática da Enfermagem Forense.

## INTRODUÇÃO

A Enfermagem Forense, reconhecida como especialidade no Brasil desde 2011^(1)^, e regulamentada pelo Conselho Federal de Enfermagem^(2)^, integra cuidados de Enfermagem e práticas forenses no atendimento a vítimas, agressores(as) e suas famílias em contextos de violência e criminalidade^(3)^. A Matriz de Competências destaca oito áreas principais, como violência sexual, sistema prisional, psiquiatria, coleta de vestígios, pós-morte, perícia e consultoria, desastres em massa e maus-tratos ao longo do ciclo da vida^(2)^.

A especialidade engloba 29 competências gerais distribuídas em seis áreas, como a avaliação e intervenção em violência e trauma, atuação como perito judicial, aplicação do Processo de Enfermagem à população prisional, e resposta a desastres e missões humanitárias, com foco na colaboração interdisciplinar. Além disso, inclui 37 competências específicas, como avaliação de riscos à saúde em vítimas de violência sexual, assistência no sistema penal e psiquiátrico, elaboração de laudos, preservação de vestígios, documentação fotográfica e atuação no pós-morte em desastres^(2)^.

Essas competências profissionais são fundamentadas em aspectos ético-legais e na assistência às pessoas em situações de violência^(4,5)^. A violência é um sério problema de saúde pública e pode manifestar-se de diversas formas, incluindo física, psicológica e/ou emocional, doméstica e sexual^(6)^, e é essencial que os profissionais de saúde estejam preparados para identificar e notificar casos por meio do Sistema de Informação de Agravos de Notificação (SINAN)^(7)^.

A atuação da Enfermagem na Atenção Primária à Saúde (APS) é vital para reconhecer sinais e sintomas de violência, mas muitos profissionais carecem de formação e habilidades para lidar com essas situações^(8)^. A falta de notificação compulsória e a necessidade de capacitação para atender essas pessoas são questões críticas que demandam estratégias educativas e políticas públicas eficientes^(9)^.

Intervenções educativas, especialmente em ambientes profissionais e universitários, são fundamentais para preparar futuros profissionais de saúde para lidar com a violência^(10,11)^. A formação deve incluir conteúdos específicos sobre violência, capacitando Enfermeiros a reconhecer e intervir em diferentes casos de violência e outras formas de agressão no âmbito forense, como estratégias eficazes para o combate e a prevenção desse fenômeno mundial^(12)^.

A escassez de estudos sobre Enfermagem Forense nas instituições de ensino no Brasil evidencia a necessidade de incorporar esses temas na formação acadêmica, a fim de promover uma prática clínica baseada em evidências de maneira mais eficaz^(13,14)^. A atuação dos Enfermeiros Forenses é fundamental para a identificação e a intervenção em casos de violência. A avaliação do conhecimento desses profissionais acerca de suas competências profissionais é essencial para aprimorar a assistência oferecida, além de contribuir de forma significativa com outras áreas, como a justiça, a educação e a segurança pública^(15)^.

### Relevância do estudo

Este estudo é um recorte da tese de doutorado de um dos autores, intitulada “Competências de enfermagem forense: avaliação do conhecimento de enfermeiros”^(16)^.

## OBJETIVOS

Analisar o conhecimento dos Enfermeiros da Atenção Primária à Saúde sobre competências em Enfermagem Forense antes e após intervenção educativa.

## MÉTODO

### Aspectos éticos

O estudo passou pela aprovação do Comitê de Ética em Pesquisa da universidade. Os profissionais foram convidados a participar da pesquisa via *e-mail* e, estando de acordo, foram direcionados automaticamente para o Termo de Consentimento Livre e Esclarecido (TCLE) via eletrônica, de acordo com a Resolução nº 466/12.

### Desenho, período e local do estudo

Este estudo de intervenção apresenta características de uma pesquisa quase experimental, utilizando pré e pós-testes sem grupo controle. Foi conduzido com Enfermeiros da APS vinculados da região do Sul de Minas Gerais. A coleta de dados ocorreu entre março e agosto de 2023.

### População ou amostra; critérios de inclusão e exclusão

O total de participantes atuantes na APS foi de 153 profissionais, sendo 125 vinculados à Estratégia Saúde da Família (ESF) e 28 às Unidades Básicas de Saúde (UBS). Para a realização do estudo, recrutou-se 96 profissionais, dos quais 42 participaram do pré-teste e 12 completaram todas as etapas da pesquisa (pré-teste, intervenção e pós-teste), compondo a amostra final do estudo. Todos os participantes possuíam no mínimo um ano de experiência, encontravam-se em exercício de suas funções durante o período da pesquisa e apresentaram disponibilidade para participar dos três momentos do estudo. Já os critérios de exclusão abrangeram aqueles que estavam em período de férias e/ou em afastamento por motivos médicos.

### Protocolo do estudo

O estudo foi realizado em três etapas: pré-teste, intervenção e pós-teste. Na primeira, avaliou-se o perfil e o conhecimento prévio por meio do “Questionário de Avaliação de Conhecimento em Enfermagem Forense na APS”, elaborado e validado pelos autores^(17)^. Ele é autoaplicável, do tipo *Likert*, com cinco opções de resposta. O questionário foi aplicado via *Google Forms* antes e após a intervenção.

A segunda etapa envolveu a intervenção educativa, a qual consistiu em um curso de extensão, teórico-prático, fundamentado na Matriz de Competências^(3)^. Ele foi estruturado em oito módulos: I: Contextualização da especialidade; II: Principais áreas de atuação no Brasil; III: Tipologias da violência e suas características; IV: Processo de Enfermagem Forense; V: Papel da Enfermagem nas Provas forenses; VI: Aspectos forenses em situações psiquiátricas; VII: Maus-Tratos, traumas e outras formas de violência; e VIII: Enfermagem Forense e os direitos civis e penais. O curso foi ministrado pelos três pesquisadores simultaneamente. Os profissionais receberam o conteúdo básico ministrado em formato digital. A carga horária do curso constituiu-se por 60 horas, sendo 40 na modalidade síncrona e 20 assíncrona, utilizou-se a Metodologia da Problematização com o Arco de Maguerez como estruturação pedagógica^(18)^. Ele foi ministrado *online,* via *Google Meet* de forma síncrona, com 10 encontros semanais de quatro horas cada, às terças-feiras. Atividades assíncronas foram realizadas às quartas-feiras através de ferramentas como *Google Forms* e *Moodle*. Além disso, os participantes receberam uma apostila com conteúdo teórico e estudos de caso. Por fim, na terceira, após a intervenção, os participantes foram reavaliados com o questionário acima citado^(17)^.

### Análise dos resultados e estatística

Para a análise e organização dos dados, utilizou-se o *software R*, versão 4.2.3. A caracterização sociodemográfica e laboral dos participantes foi realizada por meio de análises descritivas e inferenciais, tanto no pré-teste quanto no pós-teste. O conhecimento em relação às competências da área foi avaliado utilizando estatísticas descritivas e o teste exato de Fisher, com nível de significância de 5% para a comparação do antes e depois do curso de extensão.

## RESULTADOS

A análise dos dados sociodemográficos revelou predominância do sexo feminino em ambas as etapas (90,48% no pré-teste e 75,00% no pós-teste), com maior concentração (57,14% no pré-teste e 50,00% no pós-teste) etária entre 30 e 40 anos. Observou-se mudança na variável cor/raça, com maioria parda no pré-teste (69,05%) e branca no pós-teste (75,00%). Quanto ao estado civil, casados/companheiros foram maioria no pré-teste (57,14%), enquanto solteiros predominaram no pós-teste (50,00%). Houve avanço na escolaridade: 19,05% tinham ensino superior completo no pré-teste, contra 100% no pós-teste, sendo que 83,33% cursavam pós-graduação. A renda familiar manteve-se estável entre 4 a 6 salários-mínimos (52,38% no pré-teste e 50,00% no pós-teste). A crença católica prevaleceu nas duas fases (76,19% no pré-teste e 83,33% no pós-teste).

No perfil laboral, a distribuição por municípios variou, com Alfenas e Muzambinho mantendo participação nas duas fases. A experiência profissional entre 5 e 10 anos aumentou de 14,29% para 50%, indicando maior adesão de profissionais com atuação consolidada. A carga horária de trabalho foi predominantemente de 8 horas diárias (97,62% no pré-teste e 100% no pós-teste), e a maioria não possuía outro emprego. As avaliações de conhecimento entre o pré e o pós-teste mostraram variações significativas nas competências relacionadas à Enfermagem Forense, conforme será detalhado a seguir.

### A) Violência Física, Psicológica e/ou Emocional, Sexual e Doméstica/Familiar

A intervenção educativa demonstrou impacto no conhecimento em relação às diferentes tipologias de violência - física, psicológica/emocional, sexual e doméstica/familiar. Em relação à física, observou-se associação na identificação (p<0,0216), avaliação e intervenção (p<0,0063), com aumento de até 80,00% no conhecimento após o curso. Quanto à psicológica/emocional, houve avanços na identificação (p<0,0074) com 75,00% de assertividade e, na intervenção (p<0,0127), com 90,00% dos profissionais relatando maior confiança, embora 16,00% ainda tenham apresentado incertezas na avaliação. A sexual também apresentou associação, com aumento do conhecimento para identificação (p<0,0012), avaliação (p<0,0359) e intervenção (p<0,0095), com cerca de 90,00% dos profissionais se sentindo mais preparados após a capacitação. De forma semelhante, os dados sobre violência doméstica/familiar indicaram associação na identificação (p<0,0024), avaliação e intervenção (p<0,0127), com 90,00% dos participantes expressando maior segurança para atuar. De modo geral, os resultados confirmam que a capacitação fortaleceu significativamente as competências dos profissionais, especialmente nas dimensões de identificação e intervenção, destacando a importância de estratégias educativas para qualificar a atuação frente às diferentes formas de violência.

### B) Criminalidade Relacionada à Enfermagem Forense

Em relação à identificação de situações associadas à criminalidade, os resultados apontaram associação na identificação de perpetradores(as) de violência (p<0,0007), indivíduos com comportamentos violentos (p<0,0060), tentativas ou consumação de aborto ilegal (p<0,0009) e vítimas de violência dentro do sistema prisional (p<0,0043). A maioria dos profissionais (50,00%) relatou aumento da confiança e preparo após a intervenção, evidenciando que o curso ampliou sua percepção e capacidade de reconhecimento dessas situações críticas.

Quanto à avaliação de contextos forenses, antes da oferta da capacitação os profissionais relataram não terem conhecimento sobre a temática. Após a intervenção houve melhora no conhecimento (40,00%) sobre a realização de exames periciais no Instituto Médico Legal (IML), com associação no pós-teste (p<0,0006). Apesar do avanço, a avaliação indicou que há necessidade de aprofundamento em alguns conteúdos, principalmente aqueles que envolvem maior complexidade técnica e prática.

Além disso, a capacitação foi eficaz em ampliar o conhecimento sobre os procedimentos de atuação em diversas frentes (40,00%). Observou-se associação nas variáveis relacionadas ao exame pericial no IML (p<0,0031) e na atuação sobre coleta (p<0,0047), armazenamento (p<0,0051) e encaminhamento de material biológico (p<0,0043). A maioria (50,00%) demonstrou maior domínio sobre o armazenamento correto desses materiais, o que indica impacto positivo, embora os dados também apontem que práticas com maior complexidade operacional ainda requerem reforço no conteúdo.

A atuação frente ao manejo de vestígios relacionados à causa da morte (50,00%) também apresentou avanços, com associação nas etapas de coleta (p<0,0033), recolha (p<0,0007) e documentação de evidências (p<0,0019). Os resultados sugerem que os módulos sobre esse tema foram efetivos; no entanto, considerando o caráter prático das habilidades, o ensino exclusivamente remoto pode ter limitado a autoconfiança de alguns participantes, exigindo, portanto, estratégias didáticas mais interativas.

Outras áreas de intervenção que apresentaram evolução no conhecimento foram: o manejo de perpetradores(as) no sistema prisional (p<0,0014), atuação com pessoas sob custódia (p<0,0028), encaminhamento a órgãos específicos (p<0,0112) e realização de boletins de ocorrência (p<0,0185). Ainda que os ganhos tenham sido evidentes (60,00%), recomenda-se o reforço de conteúdos sobre procedimentos legais e fluxos institucionais para garantir maior domínio por parte dos profissionais.

Por fim, a capacitação demonstrou resultados quanto à elaboração de laudos periciais (p<0,0003) e à notificação de casos no SINAN (p<0,0127), sendo este último o ponto que apresentou menor avanço (75,00%). Isso sugere que, apesar do impacto da intervenção, conteúdos ligados à vigilância epidemiológica e registro oficial de casos exigem abordagens mais práticas e exemplos aplicados à realidade da APS.

### C) Legislação Brasileira em Relação à Enfermagem Forense

Os resultados indicaram associação no aumento do conhecimento sobre legislações específicas de proteção social (66,00%). Houve melhora na compreensão da Lei Maria da Penha (p<0,0109), da Lei do Feminicídio (p<0,0059), da Lei Menino Bernardo (p<0,0001), do Estatuto da Criança e do Adolescente - ECA (p<0,0088) e da Lei do Minuto Seguinte (p<0,0009). Esses dados indicam que os profissionais ampliaram sua capacidade de reconhecer os dispositivos legais que embasam a identificação e o encaminhamento de casos de violência no âmbito da APS.

Houve ainda associação no conhecimento sobre outras normativas legais de impacto na atuação prática (58,00%), como a Lei Joanna Maranhão (p<0,0001), que trata de crianças e adolescentes vítimas de violência. A notificação compulsória de violência contra a mulher (VCM) também apresentou melhoria no entendimento (p<0,0037), reforçando a importância do profissional como agente de vigilância e proteção.

Adicionalmente, 66,00% dos participantes demonstraram aumento do conhecimento sobre o Estatuto do Idoso (p<0,0205), legislação essencial para a defesa dos direitos de uma população crescente e frequentemente vulnerável, bem como sobre a função do enfermeiro como perito judicial, com associação (p<0,0001), evidenciando o impacto do curso na expansão do entendimento sobre atribuições legais específicas da especialidade.

### D) Ética e Bioética na Enfermagem Forense

Em relação à identificação e avaliação de condutas éticas como imprudência, imperícia, negligência e maus-tratos, o curso promoveu avanços principalmente na identificação da imprudência (p<0,0328) e na sua avaliação (p<0,0037), indicando maior clareza dos participantes sobre os limites da atuação segura. No entanto, embora as respostas tenham melhorado nas questões relacionadas à identificação da imperícia (75,00%), a negligência e maus-tratos, houve associação sugerindo que o conteúdo pode ter gerado reflexões, mas não o suficiente para alterar de forma expressiva o nível de conhecimento desses temas no grupo avaliado. Por outro lado, na avaliação dessas mesmas condutas (65,00%), foram encontradas associações na imperícia (p<0,0072), negligência (p<0,0078) e maus-tratos (p<0,0078), evidenciando um maior entendimento das consequências dessas falhas técnicas e comportamentais na prática profissional.

Quanto aos fundamentos legais e éticos, os participantes apresentaram melhora (66,00%) no conhecimento sobre o processo médico-legal (p<0,0043), o código de ética profissional (p<0,0183), o sigilo profissional (p<0,0308) e os conceitos gerais de ética e bioética (p<0,0205). A associação entre essas variáveis demonstra que o curso foi eficaz em promover uma compreensão mais sólida sobre os princípios que regem a conduta do enfermeiro, especialmente no manejo de situações sensíveis e juridicamente relevantes.

### E) Transtornos Mentais e Comportamentais relacionada à Enfermagem Forense

A capacitação proporcionou avanços no conhecimento (75,00%) sobre saúde mental, comportamento violento, tentativa de suicídio e uso de substâncias psicoativas - temas frequentemente subvalorizados na prática clínica cotidiana. Houve associação para avaliação (p<0,0154) e intervenção (p<0,0068) em casos de violência direcionada a si mesmo ou a terceiros.

No que tange ao suicídio, identificou-se melhorias em todas as etapas analisadas (75,00%), com associação na identificação (p<0,0400), avaliação (p<0,0246) e intervenção (p<0,0068). Quanto ao uso e abuso de álcool e outras drogas, os participantes apresentaram avanços na avaliação (p<0,0328) e intervenção (p<0,0068), evidenciando maior segurança para lidar com esses casos após o curso.

Em relação a indivíduos com comportamento violento, a concordância na identificação subiu de 50% para 83,33%, e na intervenção, de 41,67% para 58,33%, ambas com associação. Situação semelhante foi observada quanto aos perpetradores(as) com transtornos mentais, cujas taxas de concordância passaram de 33,33% para 75% na identificação e para 58,34% na intervenção, com p-valores também significativos (p<0,05).

Nos atendimentos a pessoas com transtornos mentais sob mandado judicial, destacou-se a diminuição das respostas neutras e o aumento das concordantes, demonstrando maior clareza dos profissionais, embora a evolução tenha sido mais discreta. Já nos casos de internação compulsória, observou-se progresso na identificação (de 41,67% para 58,33%) e na intervenção (de 33,33% para 41,67%), com associações que reforçam a efetividade da capacitação (p<0,05).

### F) Desastres em Massa, Catástrofes e Missões Humanitárias

A capacitação também abordou situações de urgência e emergência envolvendo acidentes automobilísticos, afogamentos, desastres em massa, ferimentos por armas e asfixia. Observou-se associação estatisticamente significativa (p<0,05) nas etapas de identificação, avaliação e compreensão das noções periciais, indicando maior segurança e preparo dos profissionais para atuar nesses contextos (80,00%). Quanto ao encaminhamento para serviços como Serviço de Atendimento Móvel de Urgência, Corpo de Bombeiros e Pronto Atendimento, cerca de 90,00% dos enfermeiros já demonstravam domínio prévio, o que se manteve no pós-teste. Apesar da variação discreta, os dados (p<0,0431) sugerem que o curso atuou como um reforço importante, consolidando práticas já estabelecidas e fortalecendo a atuação da Enfermagem da APS em cenários de emergência com implicações forenses.

### G) Relacionamento Interpessoal com Vítimas e Perpetradores(as)

A capacitação abordou temas relacionados ao acolhimento humanizado, apoio emocional e Consideração Positiva Incondicional (CPI), especialmente em contextos críticos como morte, desastres e sofrimento. O acolhimento à família diante da causa de morte (p<0,0370) e a postura de não julgamento apresentaram pouca variação entre os testes, indicando conhecimento prévio consolidado (80,00%). Por outro lado, observou-se melhora significativa no aporte emocional a vítimas de desastres (p<0,0067), evidenciando que o curso supriu lacunas iniciais. Houve também evolução no apoio aos perpetradores(as) (p<0,05), embora essa área ainda demande maior aprofundamento, devido à complexidade e estigmatização envolvidas. Quanto à CPI, os resultados demonstraram avanço (p<0,0059), reforçando que a capacitação contribuiu (66,00%) para ampliar a consciência ética e a preparação dos profissionais, mesmo que a prática ainda exija apoio institucional e formação contínua para ser plenamente aplicada.

## DISCUSSÃO

Os achados demonstram o impacto da intervenção, abordando tipologias de violência, criminalidade, aspectos forenses, legislação, transtornos mentais e comportamentais, desastres em massa, missões humanitárias e relacionamentos interpessoais. Pesquisas realizadas no Brasil^(19,20)^ e em Portugal^(21)^ ressaltam a relevância de programas de capacitação que abordem a prática forense em suas múltiplas dimensões, contribuindo para uma formação mais sólida dos profissionais. Isso se traduz em intervenções mais eficazes ao atender populações afetadas pela violência. A ampliação do conhecimento sobre as competências é essencial para que esses profissionais atuem de forma mais efetiva em situações críticas^(22)^. Essa iniciativa está alinhada às diretrizes da Organização Mundial da Saúde (OMS)^(6)^, que reconhece a violência como um grave problema de saúde pública que afeta crianças, mulheres e idosos.

O perfil sociodemográfico e laboral revela que as características da Enfermagem no Brasil é predominantemente feminina e inclui uma proporção significativa de profissionais negras e pardas, além de ser marcada por profissionais casadas, com o catolicismo como religião predominante^(23,24)^. Estudo realizado no Distrito Federal aponta que a maioria desses profissionais atua na APS há mais de 12 anos^(23)^. Essa configuração sociodemográfica pode impactar na assimilação e aplicação do conhecimento adquirido, pois indicam que mulheres nessa faixa etária apresentam maior sensibilidade a questões relacionadas à violência^(24)^.

A capacitação demonstrou impacto positivo, refletido no aumento do conhecimento quanto à intervenção em casos de violência. Esses resultados estão em consonância com um estudo realizado no Irã^(25)^, que evidenciou o efeito de programas educativos na habilidade dos profissionais em elaborar estratégias adequadas para aprimorar o conhecimento e a tomada de decisão clínica diante de pacientes forenses. A intervenção abordou diferentes tipologias de violência, favorecendo uma atuação mais assertiva tanto na identificação quanto na resposta às situações apresentadas. Tal abordagem também vai ao encontro de achados de uma pesquisa conduzida na Índia^(26)^, a qual ressalta a importância da qualificação dos profissionais para atuação e melhorias no cuidado forense.

Por outro lado, um estudo revelou desafios enfrentados por profissionais na assistência a mulheres em situação de violência. Os principais problemas identificados foram a falta de capacitação, que resulta em falhas no atendimento e assistência inadequada, além da subnotificação dos casos^(27)^. A conduta predominante observada foi a escuta qualificada, considerada uma habilidade essencial no cuidado às vítimas de violência^(27)^.

A literatura sobre intervenções em casos de violência física indica que as mulheres são as principais vítimas^(28)^. Observa-se um despreparo dos profissionais, que não apenas enfrentam desafios no tratamento das lesões físicas, mas também carecem de uma abordagem mais ampla que considere os aspectos psicológicos da violência. As intervenções devem incluir acolhimento, identificação de sinais e sintomas, além do encaminhamento para redes de apoio que integrem equipes interdisciplinares. Para melhorar essa situação, é fundamental investir em educação continuada nos serviços e incluir disciplinas que abordem essa temática nos currículos de ensino^(28,29)^.

No que se refere à avaliação e à intervenção de violência psicológica e/ou emocional, um estudo realizado na África do Sul^(30)^ e outro na Austrália^(31)^ revelam que os Enfermeiros que atuam nos serviços de saúde mental e psiquiatria forense enfrentam diferentes formas de violência, incluindo violência que geram traumas físicos e psicológicos. Os estudos citados anteriormente ressaltam que essa exposição tem um efeito psicológico negativo sobre os profissionais, afetando sua saúde mental. Para lidar com essas experiências, os profissionais utilizam diferentes estratégias, e o estudo sugere que oferecer apoio psicológico e treinamentos específicos pode ajudá-los a gerenciar melhor o impacto da violência que enfrentam no ambiente profissional.

Em relação à intervenção em violência sexual, os dados do presente estudo mostraram um impacto dos participantes relatando concordância quanto à intervenção após o curso. O resultado é relevante, uma vez que a violência sexual representa um dos maiores desafios para os profissionais, devido à sua complexidade e às implicações ético-legais e clínicas^(32)^. A pesquisa também destaca a fragilidade na formação profissional, evidenciada pela insuficiência das atuais medidas de capacitação, o que, muitas vezes, faz com que se sintam despreparados para lidar com casos de crimes sexuais^(33)^.

No âmbito da intervenção e avaliação em casos de violência doméstica e/ou familiar, o curso promoveu avanços, evidenciados pelo aumento do conhecimento sobre intervenções adequadas. Um estudo sobre a atuação forense com idosos em situações de violência^(34)^ enfatiza que os Enfermeiros generalistas frequentemente aplicam estratégias da especialidade, mesmo sem reconhecer formalmente esse conhecimento. Contudo, existem barreiras que dificultam esse atendimento, com a ausência de treinamentos adequados sendo uma das principais dificuldades^(34)^.

Além de fortalecer o conhecimento sobre criminalidade e práticas forenses, a intervenção contribuiu para a compreensão de temas como aborto ilegal, assistência ao perpetrador(a) e procedimentos periciais. Apesar do avanço sobre coleta e armazenamento de material biológico, não foi observada associação, indicando a necessidade de uma capacitação mais aprofundada e direcionada para essa temática. Quando bem recebida pelos profissionais da APS, a intervenção favorece a identificação precoce e o adequado encaminhamento dos casos, tanto dentro quanto fora da rede, ampliando a visibilidade e a efetividade dos serviços de atenção à violência. Contudo, sua implementação ainda encontra desafios, os quais estão relacionados à estrutura organizacional dos serviços municipais e à cultura assistencial vigente, que pode limitar a incorporação integral das práticas forenses na rotina da APS^(35)^.

De acordo com o estudo na Nova Zelândia^(36)^, uma das maiores dificuldades enfrentadas está na aplicação prática de técnicas periciais, o que pode justificar os resultados obtidos. Além disso, a pesquisa apresentou conhecimentos limitados sobre assistência aos pacientes forenses, preservação e coleta de evidências, legislação e/ou processos legais. As sugestões indicam que a educação forense é necessária e desejada entre profissionais dentro do ambiente clínico. Um estudo desenvolvido no Brasil^(19)^ ressalta a necessidade do conhecimento, treinamento, e participação prática do profissional habilitado ao atendimento e coleta de vestígios para fins legais no cuidado a pacientes vítimas de violência.

O impacto da capacitação sobre o conhecimento legislativo no âmbito forense, incluindo leis como a Maria da Penha e a do Feminicídio. A legislação brasileira^(37)^ sobre a proteção das mulheres tem ganhado destaque nas discussões sobre esse tema. Há uma necessidade de incluir, desde a formação continuada e o treinamento das equipes, o conhecimento das normas que garantem proteção às mulheres em situação de violência. Essa compreensão deve ser amplamente assimilada pelos profissionais, permitindo o desenvolvimento de ações efetivas de prevenção e proteção. A falta desse conhecimento pode limitar a atuação adequada dos profissionais que enfrentam essa realidade^(38)^.

A capacitação também se mostrou eficaz no conhecimento sobre ética e bioética e associação entre as variáveis. Essa área é frequentemente citada como um desafio na prática forense, pois envolve aspectos delicados como a preservação do sigilo e a condução ética de casos criminais. Um estudo realizado na Índia^(26)^ destaca que o papel da Enfermagem vai além das competências técnico-científicas. O Enfermeiro Forense, ao coletar evidências, deve agir sem preconceitos, com cautela e atenção à prevenção e redução da violência. Além disso, é essencial que desenvolva habilidades ético-legais e culturais, garantindo que não cause danos à pessoa em situação de crime.

Embora a capacitação tenha aumentado o conhecimento sobre a identificação e intervenção em casos de transtornos mentais e comportamentais, os resultados foram significativos. Tal achado pode estar relacionado à complexidade dos casos envolvendo saúde mental e forense, como destacado em estudo desenvolvido no Japão^(39)^, em que os pesquisadores observaram que em muitos casos, os Enfermeiros Psiquiátricos Forenses enfrentam dificuldades em gerenciar esses casos sem suporte contínuo, falta de treinamento especializado e um cuidado negligenciado por falta de intervenções que pudessem modificar essa realidade.

Os resultados relativos à capacitação para desastres em massa e missões humanitárias indicam um aumento no conhecimento e associação. Esse resultado é consistente com estudos que apontam a necessidade de maior ênfase em capacitações sobre desastres e emergências, áreas em que Enfermeiros muitas vezes carecem de preparo específico^(20)^. A preparação para situações extremas é crucial, como também apontado pela literatura sobre resposta a desastres^(19)^.

A análise do relacionamento interpessoal com vítimas e perpetradores(as) destacou o conhecimento positivo pré-existente entre os Enfermeiros, com aumento no conhecimento sobre suporte emocional após a capacitação. Embora os resultados tenham sido significativos, esse aspecto reforça a importância de treinamentos focados na comunicação e empatia, áreas fundamentais para a prática de Enfermagem Forense no contexto humanitário^(40)^.

Além do mais, os Enfermeiros, sendo o maior grupo de cuidadores e com constante contato com pessoas em situações de violência, desempenham um papel crucial na defesa dessas pessoas, especialmente na preservação de vestígios. Assim, é fundamental investir na formação desses profissionais para uma atuação mais abrangente e eficaz, contribuindo para um atendimento ideal a esse público^(41)^.

Portanto, a violência é um problema persistente na sociedade contemporânea e os Enfermeiros Forenses desempenham um papel crucial na sua abordagem, uma vez que possuem conhecimentos especializados para atuar em casos criminais. A prática forense abrange não apenas a análise de vítimas e perpetradores(as), mas também a assistência aos sobreviventes e familiares, ressaltando sua importância em uma resposta abrangente a esse fenômeno^(42)^.

### Limitações do estudo

Entre as limitações do estudo, destaca-se o tamanho reduzido da amostra, o que limita a generalização dos resultados. Além disso, observou-se dificuldade por parte dos profissionais da APS em compreender alguns temas, especialmente perícia criminal, legislação e desastres em massa. Apesar da oferta do curso em formato on-line e encontros semanais, a temática pode ter sido percebida como distante da prática cotidiana da APS. Embora apresentem noções básicas, ainda existem lacunas importantes em áreas como cuidado com agressores(as) e atuação legal. Esses achados reforçam a necessidade de incluir conteúdos forenses de forma contínua e estruturada na formação dos profissionais, para ampliar sua capacidade de resposta ética, técnica e legal frente a situações de maior complexidade.

### Contribuições para a área da Enfermagem, Saúde ou Política Pública

As competências em Enfermagem Forense contribuem significativamente para o enfrentamento da violência, ao capacitar profissionais da APS para identificar sinais de abuso, preservar evidências e atuar de forma ética e legal. Essa atuação fortalece a resposta integrada à violência nos serviços de saúde, melhora o atendimento às vítimas e apoia políticas públicas mais eficazes e humanizadas, promovendo impactos positivos na saúde comunitária e na proteção social.

## CONCLUSÕES

A análise do conhecimento dos enfermeiros da APS sobre Enfermagem Forense, realizada em três etapas (pré-teste, intervenção e pós-teste), demonstrou avanço significativo após a capacitação. Houve melhoria nas competências de identificação, avaliação e intervenção frente a diversas formas de violência, além de maior compreensão sobre criminalidade, ética, bioética e aspectos legais da prática forense. Os participantes também se mostraram mais preparados para atuar em contextos complexos como desastres, transtornos mentais e situações de urgência com implicações jurídicas. No entanto, persistem lacunas em temas como assistência a pacientes forenses, manejo de evidências e conhecimentos técnico-jurídicos. Esses achados evidenciam a necessidade de programas contínuos de educação voltados à formação forense dos profissionais da APS, alinhados às exigências atuais da prática ética e legal na atenção básica.

## Data Availability

Não se aplica.
